# The complete mitochondrial genome of *Pomacanthus arcuatus* (Perciformes, Pomacanthidae)

**DOI:** 10.1080/23802359.2020.1719924

**Published:** 2020-02-02

**Authors:** Liguo Yang, Yue Wang, Xuefeng Song, Na Ying, Bo Qin, Yanqing Wu

**Affiliations:** a Key Laboratory of East China Sea Fishery Resources Exploitation, Ministry of Agriculture, East China Sea Fisheries Research Institute, Chinese Academy of Fishery Sciences, Shanghai, China;; b Guangdong Provincial Key Laboratory of Marine Biotechnology, Institute of Marine Sciences, Shantou University, Shantou, China

**Keywords:** *Pomacanthus arcuatus*, Pomacanthidae, mitochondrial genome

## Abstract

*Pomacanthus arcuatus* is one of most important genera of Pomacanthidae. However, the systemically classification and taxonomic studies have so far been limited. In this study, we report the complete mitochondrial genome sequence of *P*. *arcuatus*. The mitogenome has 16,718 base pairs (54.4% A + T content) and made up of total of 37 genes (13 protein-coding, 22 transfer RNAs and 2 ribosomal RNAs), and a putative control region. This study will provide useful genetic information for future phylogenetic and taxonomic classification of Pomacanthidae.

*Pomacanthus arcuatus* belongs to the Family Pomacanthidae and the Order Perciformes, the gray angelfish (*P. arcuatus*) is a large angelfish of the family Pomacanthidae, found in the western Atlantic from New England to the vicinity of Riode Janeiro, Brazil, and also the Bahamas, the Gulf of Mexico, and the Caribbean, including the Antilles, at depths of between 2 and 30 m (Steward et al. 2009).

There is no report of the complete genome of this species *P. arcuatus*, which was developed in Shenzhen, Guangdong Province, Republic of China (N22°37′34″, E114°41′06″) in October 2018. Therefore, it is very important to characterize the complete mitogenome of this species, which can be utilized in research on taxonomic resolution, population genetic structure and phylogeography, and phylogenetic relationship. Total DNA was extracted from muscle following TIANamp Marine Animals DNA Kit (Tiangen, China), and NOVOPlasty software was used to assemble the mitogenomes, the mistake parameter was set by default (Dierckxsens et al. 2017). The samples were stored in –80 °C in Key Lab of South China Sea Fishery Resources Exploitation &Utilization, Ministry of Agriculture and Rural Affairs, South China Sea Fisheries Research Institute, Chinese Academy of Fishery Sciences, Guangzhou, China. Number is PA-1.

In this study, we obtained the complete mitochondrial genome of the *P. arcuatus*. Its mitochondrial genome has been deposited in the GenBank under accession number MN872233. For a better understanding of genetic status and the evolutionary study, we focused on the genetic information contained in the complete mitochondrial genomes of the fish.

The complete mitogenome of the *P. arcuatus* was 16,718 bp in length. The genomic organization was identical to those of typical vertebrate mitochondrial genomes, including two rRNA genes, 13 protein-coding genes, 22 tRNA genes, a light-strand replication origin (OL), and a putative control region (CR). The overall base composition was 27.9% of A, 26.5% of T, 29.2% of C, and 16.4% of G with a slight A + T bias (54.4%) like other vertebrate mitochondrial genomes. The features mentioned above were accordant with typical Pomacanthidae fish mitogenome.

For the 13 protein-coding genes, 11 genes started with ATG while only *COI* and *ND1* started with GTG. This feature was common among vertebrate mitochondrial protein-coding genes. *P. arcuatus* had two non-coding regions, the L-strand replication origin region (36 bp) locating between tRNA-Asn and tRNA-Cys, and the control region (882 bp) locating within the tRNA-Pro and tRNA-Phe. Except for eight tRNA (tRNA-Ser, tRNA-Pro, tRNA-Glu, tRNA-Tyr, tRNA-Cys, tRNA-Asn, tRNA-Ala, and tRNA-Gln) and the *ND6* gene were encoded on the L-strand, the others were encoded on the H-strand. This feature is similar to other fish mitochondrial genes. The complete mitogenome sequence had 16 s RNA (1,713 bp) and 12 s RNA (962 bp), which were located between tRNA-Phe and tRNA-Leu and separated by tRNA-Val gene. The location is same with most vertebrates that have high conservation.

To determine taxonomic status of *P. arcuatus*, we reconstructed the phylogeny of this species with other natural populations based on the *COI* gene, the phylogenetic tree showed that the *P. arcuatus* has the closer relationship with *Pomacanthus xanthometopon* (Figure 1). The phylogeny was reconstructed based on the General Time Reversible + Invariant + gamma sites (GTR + I+ G) model of nucleotide substitution using Mega7 (Kumar et al. 2016). The complete mitochondrial genome sequence of the *P. arcuatus* provided an important dataset for a better understanding of the mitogenomic diversities and evolution in fish as well as novel genetic markers for studying population genetics and species identification.

**Figure 1.**
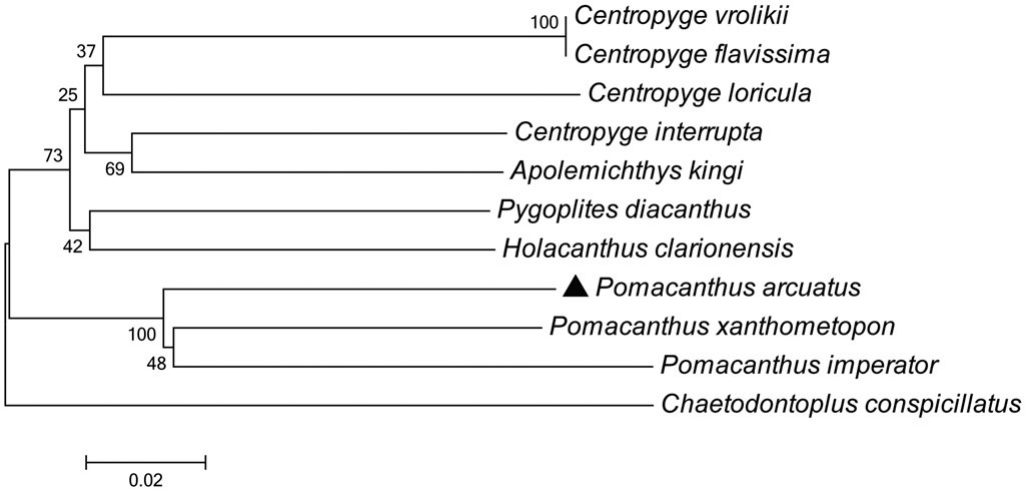
The phylogenetic relationship was estimated using the Maximum Likelihood method for the *COI* genes. Genbank accession Numbers: *Pomacanthus xanthometopon* (KP218258), *Pomacanthus imperator* (KP218259), *Apolemichthys kingi* (KP218261), *Holacanthus clarionensis* (KP218260), *Pygoplites diacanthus* (KP033453), *Centropyge interrupta* (KP018404), *Centropyge vrolikii* (NC_036949), *Centropyge flavissima* (KP973960), *Centropyge loricula* (AP006006), *Chaetodontoplus conspicillatus* (KP033452) and *Pomacanthus arcuatus* (MN872233). The numbers at the nodes are bootstrap percent probability values based on 1000 replications.
